# Aberrant Neuronal Dynamics during Working Memory Operations in the Aging HIV-Infected Brain

**DOI:** 10.1038/srep41568

**Published:** 2017-02-03

**Authors:** Tony W. Wilson, Amy L. Proskovec, Elizabeth Heinrichs-Graham, Jennifer O’Neill, Kevin R. Robertson, Howard S. Fox, Susan Swindells

**Affiliations:** 1 Department of Neurological Sciences, University of Nebraska Medical Center (UNMC), Omaha, NE, USA; 2 Department of Pharmacology and Experimental Neuroscience, UNMC,Omaha, NE, USA; 3 Center for Magnetoencephalography, UNMC, Omaha, NE, USA; 4 Department of Psychology, University of Nebraska–Omaha, NE, USA; 5 Department of Internal Medicine, Division of Infectious Diseases, UNMC, Omaha, NE, USA.; 6 Department of Neurology, University of North Carolina School of Medicine, Chapel Hill, NC, USA.

## Abstract

Impairments in working memory are among the most prevalent features of HIV-associated neurocognitive disorders (HAND), yet their origins are unknown, with some studies arguing that encoding operations are disturbed and others supporting deficits in memory maintenance. The current investigation directly addresses this issue by using a dynamic mapping approach to identify when and where processing in working memory circuits degrades. HIV-infected older adults and a demographically-matched group of uninfected controls performed a verbal working memory task during magnetoencephalography (MEG). Significant oscillatory neural responses were imaged using a beamforming approach to illuminate the spatiotemporal dynamics of neuronal activity. HIV-infected patients were significantly less accurate on the working memory task and their neuronal dynamics indicated that encoding operations were preserved, while memory maintenance processes were abnormal. Specifically, no group differences were detected during the encoding period, yet dysfunction in occipital, fronto-temporal, hippocampal, and cerebellar cortices emerged during memory maintenance. In addition, task performance in the controls covaried with occipital alpha synchronization and activity in right prefrontal cortices. In conclusion, working memory impairments are common and significantly impact the daily functioning and independence of HIV-infected patients. These impairments likely reflect deficits in the maintenance of memory representations, not failures to adequately encode stimuli.

Cognitive impairments are the central feature of HIV-associated neurocognitive disorders (HAND), which currently affect 35–70% of all HIV-infected patients, including those treated with combination antiretroviral therapy^1^,^2^,^3^,^4^,^5^,^6^. Despite this high prevalence, diagnosing HAND is often difficult, requiring cognitive testing and exclusion of opportunistic infections, psychiatric disorders, drug toxicities, brain tumors, and other possibly contributing factors. Notably, there are currently no diagnostic tests or biomarkers that can identify HAND and the underlying neural mechanisms are not fully understood. However, the brain is known to be a major HIV reservoir that is not well penetrated by antiretroviral drugs^7^, and this likely contributes to the persistently high prevalence of HAND in the modern era.

Impairments in working memory are common in HAND, especially in older patients, and are widely thought to contribute to other higher-level deficits in reasoning and decision making, which in-turn have a major impact on the patient’s daily functioning and independence^1^,^3^,^6^,^8^,^9^,^10^,^11^,^12^,^13^. While the existence of these deficits in both verbal and spatial domains has been recognized, their precise origins remain a longstanding debate. Some neuropsychological studies of HIV-infected patients have indicated that these working memory impairments arise from encoding deficiencies, whereas others suggest that central executive processes are abnormal^9^. Functional neuroimaging investigations have tended to support both views, although their conclusions remain tentative as deficits in encoding stimuli would necessarily affect the maintenance of such memory representations, and studies to date have not been able to temporally distinguish encoding and maintenance periods^9^,^12^,^13^,^14^. Furthermore, functional imaging studies in HIV-infected patients have often reported deficits in both primary sensory regions and higher-order frontal and prefrontal cortices, which again conflates the encoding and maintenance operations serving working memory function^12^,^13^,^14^,^15^,^16^,^17^,^18^. In this study, we examined working memory processing using an advanced, dynamic functional mapping approach based on magnetoencephalographic (MEG) imaging in a cohort of HIV-infected older adults and a demographically-matched sample of uninfected controls. Our primary hypothesis was that encoding processes would be preserved in HIV-infected patients, but that memory maintenance and rehearsal operations would be disturbed.

## Results

**Figure 1:**
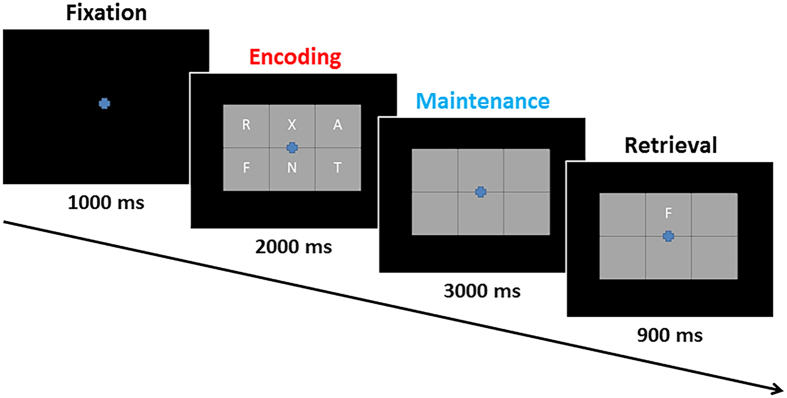
Working Memory Paradigm. Each trial consisted of four phases. (1) A fixation phase which lasted for 1.0 s, (2) an encoding phase (red text) in which a grid containing six letters was presented for 2.0 s, (3) a maintenance phase (blue text) in which the letter stimuli were removed from the grid for 3.0 s, and (4) a retrieval phase in which one probe letter was presented for 0.9 s and the participant responded as to whether it had been included in the previous encoding set.

Eighteen HIV-infected older adults were individually-matched to 18 healthy controls in regards to age, sex, ethnicity, and handedness. All 36 participants were seated in a nonmagnetic chair during the MEG recording and completed a visual working memory task. Briefly, participants were instructed to fixate on a crosshair presented centrally for 1.0 s. A grid containing six letters was then presented for 2.0 s (encoding). These letters then disappeared from the grid and 3.0 s later (maintenance phase) a single “probe” letter appeared for 0.9 s (retrieval phase; Fig. 1). Participants were instructed to respond with a button press as to whether the probe letter was one of the six letters previously presented. Each trial lasted 6.9 s, including a 1.0 s pre-stimulus fixation, and each participate completed approximately 128 trials.

Of the 36 total participants, two HIV-infected and one uninfected participant were excluded from all analyses due to poor MEG signal quality. Mean age was 57.06 years (range: 50–70) in the remaining HIV-infected group and 58.65 years (range: 50–70) in the control group. This difference was not significant (*p* = 0.52). The mean duration of HIV diagnosis was 16.69 years (range: 10–24) and the mean CD4 + T-cell count was 759 cells/mm^3^ (range: 280–1391). Eight of the 16 HIV-infected patients scored in the impaired range on at least two domains of the neurocognitive battery^1^, and the uninfected controls performed significantly better (mean accuracy: 82.4%) than HIV-infected patients (75.9%) on the working memory MEG task, t(31) = 2.04 (*p* < 0.05).

### Sensor-Level Analysis

**Figure 2:**
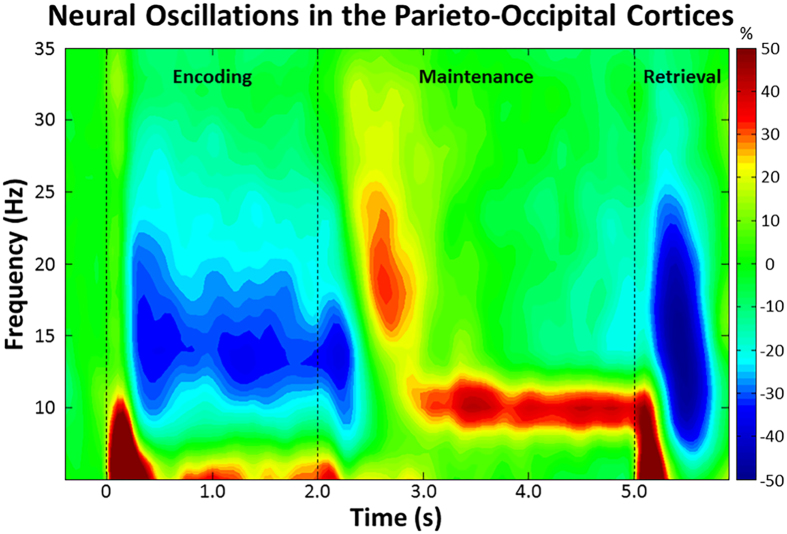
Time-frequency spectrogram with time (s) shown on the x-axis and frequency (Hz) denoted on the y-axis. Percent power change was computed by dividing the mean power of each time-frequency bin by the respective bin’s baseline power (−0.4 to 0 s) and multiplying this value by 100. The color legend is displayed to the right. Data represent a group-averaged peak sensor, collapsed across HIV-infected and uninfected groups, located near the parieto-occipital cortex. As shown, strong alpha/beta desynchronization was observed shortly after the encoding grid was presented, and this evolved into a narrower alpha synchronization during maintenance.

Statistical analyses of the MEG time-frequency spectrograms revealed a significant cluster of alpha/low-beta (9–16 Hz) oscillatory activity in sensors over posterior and left hemispheric regions (*p* < 0.001, corrected; Fig. 2). This activity began 0.2 s after onset of the encoding grid, was sustained throughout the encoding phase, and terminated at 2.5 s (i.e., 0.5 s into the maintenance phase). Additionally, a significant cluster of alpha (9–12 Hz) activity was observed during the maintenance phase (*p* < 0.001, corrected; Fig. 2). This alpha activity began at 3.0 s, was sustained throughout the remainder of the maintenance phase, and sharply dissipated early in the retrieval phase. Since these responses spanned across most of the encoding and maintenance phases, respectively, we split them into 0.4 s non-overlapping time bins and separately imaged each time-frequency window in each participant.

### Dynamic Functional Imaging Analysis

**Figure 3:**
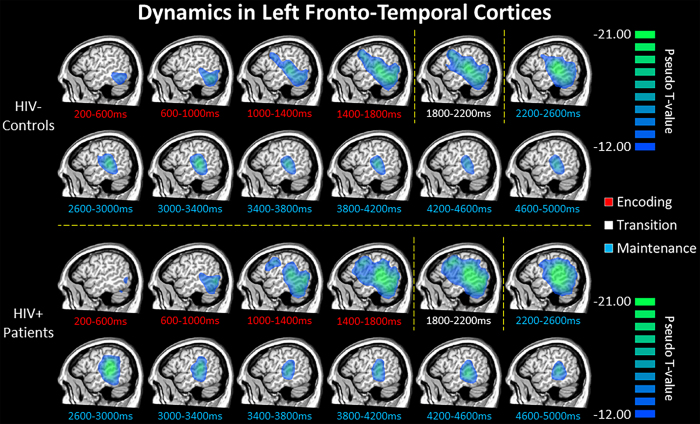
Group mean beamformer images (pseudo-t) for uninfected older adults (top) and demographically-matched HIV-infected patients (bottom) are displayed across all time bins spanning the encoding (red labels) and maintenance (blue labels) phases. Across both groups, there was a strong sustained decrease in alpha/beta oscillatory activity in left fronto-temporal cortices throughout most of the encoding and maintenance phases. This decrease began in posterior regions early in the encoding phase, spread anterior and superior to include language-related areas in the left supramarginal and prefrontal cortices during the second half of encoding and early maintenance period, then dissipated slightly throughout the remainder of the maintenance phase.

To evaluate the brain dynamics serving working memory performance, we initially examined the time course of activity in each group (Fig. 3). These data indicated a strong decrease in alpha/beta activity beginning in the early encoding phase in the bilateral occipital cortices, which rapidly spread to left superior temporal regions, supramarginal, and inferior frontal gyri. Such decreases in alpha/beta activity were sustained throughout the encoding phase in left fronto-temporal cortices, with the frontal activity starting to dissipate early in the maintenance phase. Meanwhile, alpha activity in the left superior temporal cortices extended throughout the maintenance phase. Overall, the spatiotemporal dynamics of left fronto-temporal activity were similar in HIV-infected and uninfected older adults (Fig. 3).

**Figure 4:**
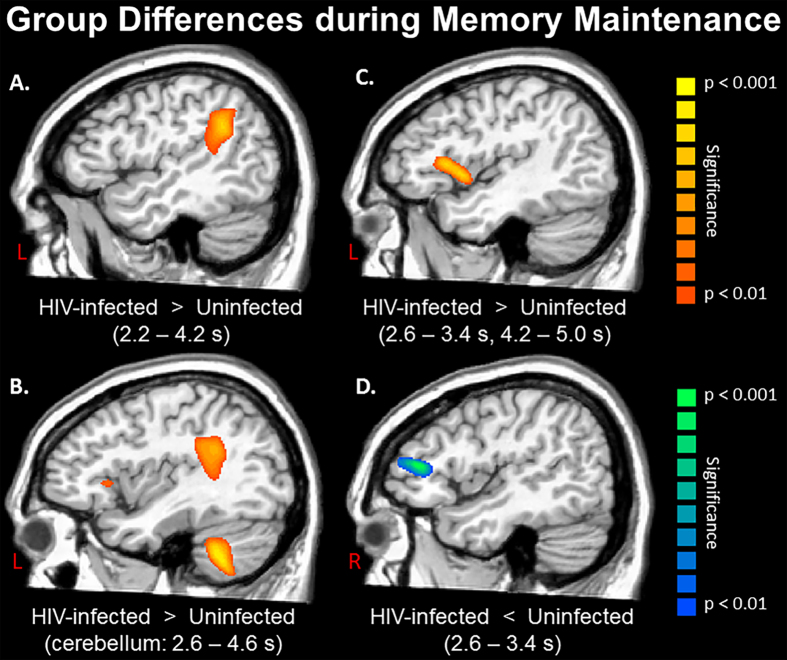
Statistical parametric maps showing group differences between HIV-infected patients and uninfected controls during the maintenance period. Time windows with significant effects are listed in the parentheses under each image. (**A**) HIV-infected patients exhibited significantly stronger alpha decreases (hyper-activation) in the left supramarginal region from 2.2 to 4.2 s after the onset of the encoding grid, which corresponds to the majority of the maintenance period. (**B**) Stronger alpha decreases in HIV-infected patients were also observed in the left cerebellum from 2.6 to 4.6 s. The group differences in the supramarginal (**A**) and left inferior frontal gyrus (IFG; panel C) can also be seen in this image. (**C**) The same pattern of stronger alpha decreases (hyper-activation) in HIV-infected patients was observed in the left IFG from 2.6 to 3.4 s and from 4.2 to 5.0 s. (**D**) Uninfected controls exhibited stronger alpha decreases in the right IFG, consistent with healthy aging studies showing recruitment of homologue cortices in the right hemisphere to aid in ongoing processing. All maps have been thresholded at (*p* < 0.01, corrected). Panels A–C depict the left hemisphere and panel D shows the right hemisphere.

**Figure 5:**
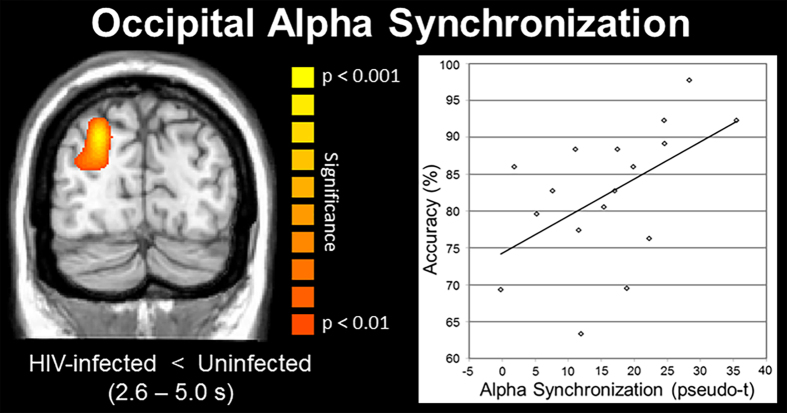
Statistical parametric map (SPM) showing group differences in alpha synchronization in occipital cortices and the accuracy correlation in uninfected controls. (**A**) Uninfected controls exhibited significantly stronger alpha synchronization in occipital cortices from 2.6 to 5.0 s after the onset of the encoding grid, which corresponds to the vast majority of the maintenance period. (**B**) The amplitude of alpha synchronization in occipital cortices was significantly correlated with accuracy in the working memory MEG task in uninfected controls (*p* = 0.03), but not HIV-infected patients. The SPM has been thresholded at (*p* < 0.01, corrected) and is displayed following radiological convention.

As for group differences, as hypothesized, encoding appeared to be relatively preserved in HIV-infected patients, while maintenance operations were severely disturbed. In sum, no significant group differences were observed in any time bin during the encoding period, yet such differences emerged immediately during memory maintenance and were generally sustained. Specifically, HIV-infected patients exhibited a significantly stronger decrease in alpha activity (i.e., hyper-activation) in the left supramarginal gyrus starting in the first maintenance period (2.2–2.6 s). This stronger recruitment was sustained into the 2.6–3.4 s time periods and also included areas of the left inferior frontal gyrus (IFG; i.e., Broca’s area) and the left cerebellum (all *p*’s < 0.01, corrected; Fig. 4). Interestingly, controls showed stronger alpha decreases in the homologue right IFG during these same time periods, as well as significantly stronger alpha synchronization in the right occipital cortices (all *p*’s < 0.01, corrected). With the exception of the IFG (left and right), the same pattern of group differences extended through the next two periods (3.4–4.2 s), with HIV-infected patients exhibiting a significantly stronger reduction in alpha activity in the left supramarginal gyrus and left cerebellum, while controls had greater alpha synchronization in both right and left occipital cortices (all *p*’s < 0.01, corrected; Fig. 5). During the next time period (4.2–4.6 s), differences in the left supramarginal and occipital dissipated, those in right occipital and left cerebellum were maintained, and new differences emerged in the left hippocampal region and reemerged in the left IFG (i.e., hyper-activation in HIV-infected patients; all *p*’s < 0.01, corrected). Finally, group differences in the left IFG and right occipital extended into the last maintenance time period (4.6–5.0 s), with HIV-infected patients having stronger decreases in left IFG and uninfected controls having stronger synchronization in the right occipital cortices (both *p*’s < 0.01, corrected).

### Neurocognitive and MEG Correlations

Pearson-correlations were conducted using the functional domain composite scores from the neuropsychological battery that was administered to patients, the working memory MEG task results, and the peak voxel values from regions where group differences were observed. These analyses indicated that performance on the MEG task was significantly correlated with global neuropsychological function (r(16) = 0.70, *p* = 0.003), memory (r(16) = 0.495, *p* = 0.05), attention (r(16) = 0.56, *p* = 0.03), speed of processing (r(16) = 0.53, *p* = 0.03), and motor function (r(16) = 0.58, *p* = 0.02) in HIV-infected patients. The strength of left supramarginal alpha activity during maintenance was also strongly correlated with global neuropsychological function (r(16) = −0.68, *p* = 0.004), performance on the MEG working memory task (r(16) = −0.62, *p* = 0.01), and marginally with attention and memory composite scores in the patient group. Interestingly, performance on the MEG working memory task was correlated with the strength of right IFG alpha activity (r(17) = −0.59, *p* = 0.01) and right occipital alpha synchronization (r(17) = 0.52, *p* = 0.03; Fig. 5) during maintenance in controls, but neither of these relationships were present in the HIV-infected patients (right IFG alpha: r(16) = 0.28, *p* = 0.29; right occipital alpha synchronization: r(16) = 0.05, *p* = 0.86).

## Discussion

Working memory dysfunction in HIV-infected patients has been widely reported, but the origin of these deficits has remained unknown. In this study, we utilized a dynamic functional mapping technique to determine whether encoding and/or maintenance operations were impaired, and to identify the critical brain regions. Our results confirmed that HIV-infected patients had working memory deficits (performance), and indicated that encoding operations were preserved while memory maintenance was aberrant, with dysfunction in occipital, fronto-temporal, hippocampal, and cerebellar cortices. Performance on the working memory MEG task and neuropsychological assessments were tightly linked to the strength of alpha activity in the left supramarginal region of HIV-infected patients, whereas MEG task performance in uninfected controls covaried with occipital alpha synchronization and activity in the right IFG.

Our most critical finding was that the neural processes serving encoding were preserved in HIV-infected patients, while those serving maintenance were clearly abnormal. Initially, HIV-infected patients exhibited stronger alpha decreases compared to uninfected controls in the left supramarginal, and this same pattern emerged in the left IFG and left cerebellum during the next time window and was sustained for 0.8 s. Studies using combined EEG/fMRI methods have connected alpha decreases in these brain regions to increased activation in the fMRI sense^19^,^20^, thus the current data indicate hyper-activation in HIV-infected patients during memory maintenance. Interestingly, during this same time window, controls had stronger alpha decreases in the right IFG relative to HIV-infected patients. Previous studies of healthy aging have shown that older adults recruit homologue regions in the right hemisphere to support working memory (and other) processes^21^,^22^,^23^,^24^,^25^, and our findings likely reflect that patients with HIV were unable to utilize these additional regions to compensate for ongoing processing. This view is supported by our finding of a significant correlation between accuracy in the MEG working memory task and neural activity levels in the right IFG of uninfected controls, but not patients. Group differences in the left and right IFG dissipated near the middle of the maintenance period, while those in the left supramarginal remained. This pattern reversed during the final 0.8 s of maintenance, with the hyper-activation in the left supramarginal dissipating and that in the left IFG reemerging in HIV-infected patients. This reversal may indicate reverberatory processing in left supramarginal and IFG cortices, which have been connected to the phonological loop and executive processing, respectively, in a popular working memory model^26^,^27^.

We also observed significantly stronger alpha synchronization in the occipital cortices of uninfected controls throughout most of the maintenance period. Many neurophysiological studies have reported similar parieto-occipital alpha synchronization during memory maintenance in healthy young and older adults^21^,^28^,^29^,^30^,^31^,^32^. An influential theory posits that such alpha activity reflects inhibition of the dorsal visual stream, which functions to protect items (e.g., letters) that are being retained in more anterior regions (e.g. Broca’s area) from being disturbed by incoming visual information^29^,^30^,^31^,^32^. Consonant with this, we propose that the significantly stronger alpha synchronization in uninfected controls reflects greater inhibition of the visual stream and thus, more effective protection of memory representations. Our behavioral data supports this interpretation, as HIV-infected patients had poor working memory performance and reduced alpha synchronization, while controls had significantly better task performance, stronger alpha synchronization, and a significant correlation between the two variables. Potentially, such alpha synchronization in occipital cortices is driven by top-down modulation from left fronto-temporal cortices, and thus could be aberrant secondary to the hyper-activation in HIV-infected patients. However, this hypothesis will need to be directly vetted in future studies using functional connectivity or related analyses.

Before closing, it is important to acknowledge some limitations. First, some of our patients had a history of drug or alcohol use, which may have had a long-term impact on cortical physiology that is not directly related to HIV. We also studied only older adults with HIV and our findings cannot be generalized to younger patients who have been infected for a shorter period of time. Lastly, our sample size was relatively modest, although it is comparable to many MEG studies in psychiatry and neurology. Future studies should use larger samples and evaluate the effects of age and infection duration. Of note, there is evidence that MEG measures have excellent test-retest reliability after ~24 weeks in both HIV-infected patients and controls^33^. To close, we used a dynamic functional brain mapping technique to determine whether encoding and/or maintenance operations underlie working memory deficits in HIV-infected patients, and to identify the critical brain regions. Our data indicated that memory maintenance was disturbed, as HIV-infected patients exhibited hyper-activation in left fronto-temporal cortices, hypo-activation in the right IFG, and reduced alpha synchronization in occipital cortices relative to demographically-matched uninfected controls. These findings are consistent with neuropsychological studies and previous fMRI studies of attention and working memory in HIV-infected patients, which also showed hyper-activation in prefrontal regions^9^,^12^,^13^,^14^,^34^. However, the current data provide critical time course information that enabled us to uniquely conclude that deficits were specific to memory maintenance, as well as identify occipital alpha deficits and abnormalities in other brain regions that had not been previously described.

## Materials and Methods

### Participant Selection

We evaluated 18 HIV-infected adults (4 females) and 18 uninfected healthy controls (5 females). Controls were individually-matched to patients in regards to age, sex, ethnicity, and handedness. All HIV-infected participants were receiving effective combination antiretroviral therapy and all but one had undetectable viremia (<20 copies/mL). Exclusionary criteria included any pre-existing major psychiatric or neurological disorder, active brain infection (except HIV-1), presence of brain neoplasm or lesion, history of head trauma, current substance abuse, and the MEG Laboratory’s standard exclusion criteria (e.g., dental braces, metal implants, etc.). Written informed consent was obtained from each participant following the guidelines of the University of Nebraska Medical Center’s Institutional Review Board, who reviewed and approved the study protocol. All methods were carried out in accordance with relevant guidelines and regulations.

### Neuropsychological Assessments

All patients underwent a battery of neuropsychological testing; published normative data were used for comparison purposes^35^. This battery adhered to the recommendations of the Frascati consensus^1^ and assessed multiple functional domains, including gross (timed gait) and fine motor (grooved pegboard), language (WRAT 4 reading), verbal learning and memory (Hopkins Verbal Learning Test – Revised), speed of processing (Trailmaking-A, digit symbol), attention and working memory (Paced Auditory Serial Addition Task), and executive functioning (verbal fluency, Stroop, and Trailmaking-B). Raw scores were transformed to demographically-adjusted z-scores prior to computing composite scores for each functional domain (e.g., motor). In total, seven composite scores were computed and used for the assessment of HAND and neurobehavioral correlation analyses with the neuroimaging data.

### Experimental Paradigm

During the MEG session, participants were seated in a nonmagnetic chair and instructed to fixate on a crosshair presented centrally for 1.0 s. A grid containing six letters was then presented for 2.0 s (encoding). These letters then disappeared from the grid and 3.0 s later (maintenance phase) a single “probe” letter appeared for 0.9 s (retrieval phase; Fig. 1). Participants were instructed to respond with a button press as to whether the probe letter was one of the six letters previously presented. Each trial lasted 6.9 s, including a 1.0 s pre-stimulus fixation. Each participant completed 128 trials and the task lasted approximately 14 minutes. This same task was previously used in two normative studies and three other clinical studies by our group^21^,^28^,^36^,^37^,^38^.

### MEG Data Acquisition and sMRI Coregistration

Recordings were conducted in a magnetically-shielded room with active shielding engaged. With an acquisition bandwidth of 0.1–330 Hz, neuromagnetic responses were sampled continuously at 1 kHz using an Elekta MEG system with 306 magnetic sensors (Elekta, Helsinki, Finland). MEG data from each participant were individually-corrected for head motion and subjected to noise reduction using the signal space separation method with a temporal extension^39^. Each participant’s MEG data were then coregistered with their structural T1-weighted MRI data using BESA MRI (Version 2.0; BESA GmbH, Gräfelfing, Germany). These neuroanatomic images were acquired with a Philips Achieva 3T X-series scanner using an eight-channel head coil and a 3D fast field echo sequence with the following parameters: TR: 8.09 ms; TE: 3.7 ms; field of view: 24 cm; matrix: 256 × 256; slice thickness: 1 mm with no gap; in-plane resolution: 0.9375 ×  0.9375 mm; sense factor: 1.5. The structural MRI volumes were aligned parallel to the anterior and posterior commissures and were transformed into standardized space after source imaging (i.e., beamforming)^40^,^41^.

### MEG Pre-Processing, Time-Frequency Transformation, and Statistics

Cardio-artifacts were removed from the data using signal-space projection, which was accounted for during source reconstruction^42^. The continuous magnetic time series was divided into epochs of 6.9 s duration, with the baseline being defined as −0.4 to 0.0 s before initial stimulus onset (Fig. 1). Epochs containing artifacts were rejected based on a fixed threshold method, supplemented with visual inspection. Artifact-free epochs were transformed into the time-frequency domain using complex demodulation with a resolution of 1.0 Hz and 50 ms, and the resulting spectral power estimations per sensor were averaged over trials to generate time-frequency plots of mean spectral density. These sensor-level data were normalized by dividing the power value of each time-frequency bin by the respective bin’s baseline power, which was calculated as the mean power during the −0.4 to 0.0 s time period.

The time-frequency windows used for imaging were determined by statistical analysis of the sensor-level spectrograms across the entire array of gradiometers during the five-second “encoding” and “maintenance” time window. Each data point in the spectrogram was initially evaluated using a mass univariate approach based on the general linear model (GLM). To reduce the risk of false positive results while maintaining reasonable sensitivity, a two stage procedure was followed to control for Type 1 error^43^,^44^. In the first stage, one-sample *t*-tests were conducted on each data point and the output spectrogram of t-values was thresholded at *p* < 0.05 to define time-frequency bins containing potentially significant oscillatory deviations across all participants. In stage two, time-frequency bins that survived the threshold were clustered with temporally and/or spectrally neighboring bins that were also significant at the (*p* < 0.05) threshold, and a cluster value was derived by summing all of the *t*-values of all data points in the cluster. Nonparametric permutation testing was then used to derive a distribution of cluster-values and the significance level of the observed clusters (from stage one) were tested directly using this distribution^28^,^43^,^44^,^45^. For each comparison, at least 10,000 permutations were computed to build a distribution of cluster values. Based on these analyses, the time-frequency windows that contained significant oscillatory events across all participants during the encoding and maintenance phases (see Results) were subjected to the beamforming analysis.

### MEG Source Imaging and Statistics

Cortical networks were imaged through an extension of the linearly constrained minimum variance vector beamformer^46^,^47^,^48^, which employs spatial filters in the frequency domain to calculate source power for the entire brain volume. The single images are derived from the cross spectral densities of all combinations of MEG gradiometers averaged over the time-frequency range of interest, and the solution of the forward problem for each location on a grid specified by input voxel space. Following convention, we computed noise-normalized source power per voxel in each participant using active (i.e., task) and passive (i.e., baseline) periods of equal duration and bandwidth. Such images are typically referred to as pseudo-t maps, with units (i.e., pseudo-t) that reflect noise-normalized power differences per voxel. All source imaging used the Brain Electrical Source Analysis (BESA) software (Version 6.1; GmbH, Gräfelfing, Germany).

Normalized source power was computed for the selected time-frequency bands over the entire brain volume per participant at 4.0 × 4.0 × 4.0 mm resolution. Preceding statistical analysis, each participant’s functional images were transformed into standardized space using the transform that was previously applied to the structural images and spatially resampled^49^. The resulting 3D maps of brain activity were averaged across participants in each group to assess the neuroanatomical basis of significant oscillatory responses identified through the sensor-level analysis. In addition, these images were statistically evaluated using a mixed effects, mass univariate approach based on the GLM. The effect of group (i.e., HIV-infected/uninfected) was determined using two-tailed independent-samples *t*-tests per time-frequency bin. All output statistical maps were displayed as a function of alpha level, thresholded at *p* < 0.01, and adjusted for multiple comparisons using a spatial extent threshold (i.e., cluster restriction; *k* = 80) based on the theory of Gaussian random fields^50^.

## Additional Information

**How to cite this article**: Wilson, T. W. *et al*. Aberrant Neuronal Dynamics during Working Memory Operations in the Aging HIV-Infected Brain. *Sci. Rep.* **7**, 41568; doi: 10.1038/srep41568 (2017).

**Publisher's note:** Springer Nature remains neutral with regard to jurisdictional claims in published maps and institutional affiliations.
